# Ornithine Aminotransferase, an Important Glutamate-Metabolizing Enzyme at the Crossroads of Multiple Metabolic Pathways

**DOI:** 10.3390/biology6010018

**Published:** 2017-03-06

**Authors:** Antonin Ginguay, Luc Cynober, Emmanuel Curis, Ioannis Nicolis

**Affiliations:** 1 Clinical Chemistry, Cochin Hospital, GH HUPC, AP-HP, 75014 Paris, France; antonin.ginguay@gmail.com; 2 Laboratory of Biological Nutrition, EA 4466 PRETRAM, Faculté de Pharmacie, Université Paris Descartes, 75006 Paris, France; 3 Laboratoire de biomathématiques, plateau iB², Faculté de Pharmacie, Université Paris Descartes, 75006 Paris, France; emmanuel.curis@parisdescartes.fr (E.C.); ioannis.nicolis@parisdescartes.fr (I.N.); 4 UMR 1144, INSERM, Université Paris Descartes, 75006 Paris, France; 5 UMR 1144, Université Paris Descartes, 75006 Paris, France; 6 Service de biostatistiques et d’informatique médicales, hôpital Saint-Louis, Assistance publique-hôpitaux de Paris, 75010 Paris, France; 7 EA 4064 "Épidémiologie environnementale: Impact sanitaire des pollutions", Faculté de Pharmacie, Université Paris Descartes, 75006 Paris, France

**Keywords:** ornithine aminotransferase, glutamate, ornithine, gyrate atrophy

## Abstract

Ornithine δ-aminotransferase (OAT, E.C. 2.6.1.13) catalyzes the transfer of the δ-amino group from ornithine (Orn) to α-ketoglutarate (aKG), yielding glutamate-5-semialdehyde and glutamate (Glu), and vice versa. In mammals, OAT is a mitochondrial enzyme, mainly located in the liver, intestine, brain, and kidney. In general, OAT serves to form glutamate from ornithine, with the notable exception of the intestine, where citrulline (Cit) or arginine (Arg) are end products. Its main function is to control the production of signaling molecules and mediators, such as Glu itself, Cit, GABA, and aliphatic polyamines. It is also involved in proline (Pro) synthesis. Deficiency in OAT causes gyrate atrophy, a rare but serious inherited disease, a further measure of the importance of this enzyme.

## 1. Introduction

Ornithine δ-aminotransferase (E.C. 2.6.1.13; OAT), or ornithine δ-transaminase, is an enzyme found in almost all eukaryotic organisms, from protozoans to humans, and from fungi to higher plants. It is strongly conserved among these organisms. In humans, OAT deficiency causes gyrate atrophy, a serious inherited disease. This suggests that OAT plays an important role in metabolism. In most situations, it uses l-ornithine (Orn) as a substrate, with l-glutamate (Glu) as an end product; OAT therefore belongs to what is termed the “glutamate crossway” [1]. At first sight, the importance of OAT may appear to be related to the roles of Glu in metabolism (bioenergetics, nitrogen flux, acid-base homeostasis), like the major enzymes involved in Glu metabolism (e.g., aspartate aminotransferase (ASAT), alanine aminotransferase (ALAT), and glutamate dehydrogenase (GDH)).

**Figure 1:**
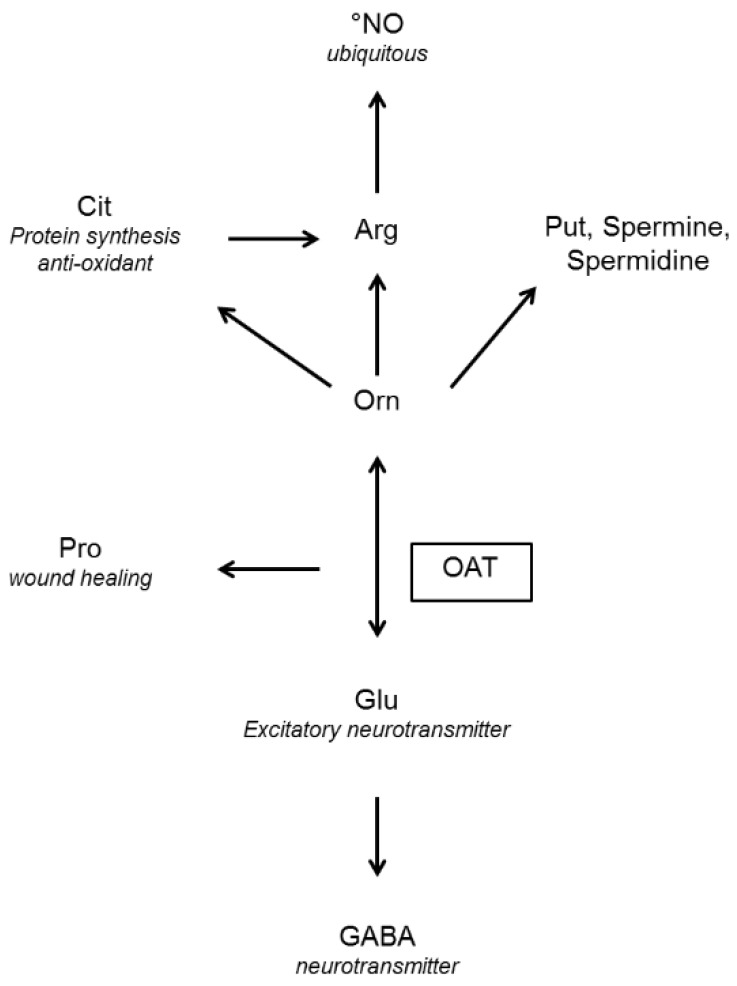
End products of the reaction mediated by ornithine (Orn) aminotransferase (OAT) and their functions (in italics) GABA: γ-aminobutyric acid, Glu: glutamate, Pro: proline, Arg: arginine, Cit: citrulline, Put: putrescine, NO: nitric oxide.

This paper summarizes current knowledge on OAT, in particular in the light of recent advances in large scale analysis, and of clinical experience, and argues that viewing the role of OAT as limited to glutamate-related energy metabolism, especially in mammals, probably misses other important functions of OAT. This is evident from an examination of the pathways relating glutamate and ornithine to several key molecules in cell metabolism and “fate” regulation (Figure 1); these functions are briefly summarized here—a detailed description lies outside the scope of this paper, but interested readers can find information in recent reviews [2,3,4,5]. To present the state of knowledge on OAT, we will first describe the physical, chemical and biological characteristics of the OAT reaction, and then go on to consider the involvement of OAT in pathological states or diseases. This systematic overview will also highlight needs for further studies of this enzyme and its regulation.

### 1.1. Physical And Chemical Aspects of the Oat-Mediated Reaction

#### 1.1.1. Reaction

**Figure 2:**
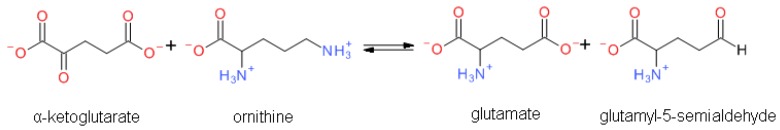
Overview of the reaction catalyzed by OAT.

OAT catalyzes the conversion of Orn into glutamyl-5-semi-aldehyde (GSA) and vice versa, using aKG and Glu as cosubstrates (Figure 2). This reaction has an apparent equilibrium constant, *K* = $\frac{\left[\mathrm{Glu}\right]\left[\mathrm{GSA}\right]}{\left[\mathrm{Orn}\right]\left[\mathrm{aKG}\right]}$, that favors the degradation of Orn: experimentally, *K* lies between 50 and 70 at 25 °C [6] and pH 7.1. Even so, when Orn is at a very low concentration, or GSA in large excess, the reaction can proceed towards Orn synthesis, which has indeed been demonstrated in vitro by Strecker et al. [6]. OAT can thus catalyze the reaction in either direction, depending on the relative amounts of each substrate present, and their use by subsequent enzymes. This observation is the first indication that OAT stands at the crossroads of several metabolic pathways.

#### 1.1.2. A Key Feature: Spontaneous Cyclization of GSA

**Figure 3:**
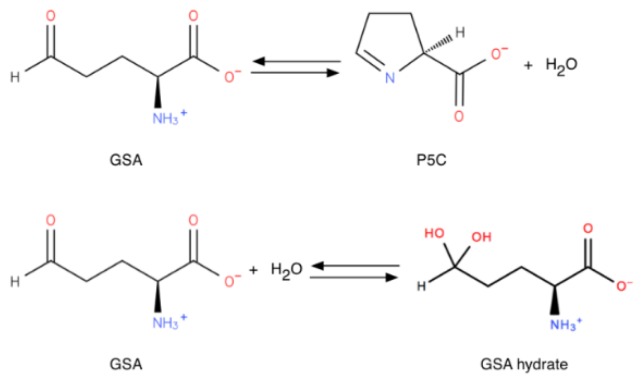
Cyclization and hydration of glutamate-5-semi-aldehyde (GSA). P5C: pyrroline-5-carboxylate.

Since GSA contains both an aldehyde and an amine function, internal imination can occur (Figure 3), leading to a chemical equilibrium with the cyclic form (*S*)-Δ^1^-pyrroline-5-carboxylate (P5C). In addition, the aldehyde can react with water to give a GSA hydrated form, as shown in Figure 3. It has been suggested since the 1930s that the cyclic forms of these types of compounds are favored for entropic reasons, as is often the case for similar reactions (almost all oses), at least at physiological pH [7,8], but the exact equilibrium point is difficult to establish because P5C undergoes polymerization [9]. For a similar compound, Δ^1^-pyrroline-2-carboxylate (P2C), a dependence on pH has been demonstrated, with the cyclic form being the only detectable one at pH above 7 [10]. Despite problems with stability the dependence of the opening of the cyclic form of P5C on pH has been accomplished by nuclear magnetic resonance (NMR) [11], using deuterated water. The two equilibria depend on pH: At pD below 6.2 the free aldehyde GSA and the hydrated gem-diol linear forms predominate, the free aldehyde increasing over the hydrated gem-diol as the pH is lowered. The free aldehyde becomes negligible at pD above 4.9. At pD 6.2 a sharp transition is detected in the NMR signal, and the cyclic P5C imine form becomes predominant above neutral pH. Thus, in the mitochondrion matrix, where the pH is around 7.8, the GSA produced by the OAT-mediated ornithine catabolism cyclizes spontaneously towards P5C, making the reaction almost irreversible in vitro [12]. The computed [13] p*K* for the amine group protonation is 9; thus at pH below 7.2, the -NH_2_ group is protonated and is not available for the imination cyclization. However, since the reaction reaches equilibrium quickly, even a minimal amount of linear GSA can be used by OAT for the reverse direction, and drive the decyclization of P5C into GSA. In addition, the mitochondrion matrix is a far more complex medium than an aqueous solution, with a rather low water content; hence the equilibrium between GSA, hydrated GSA and P5C may be quite different from that observed in water.

This equilibrium between GSA and P5C also places OAT at a metabolic crossroads, P5C opening the way to proline (Pro) and related metabolic pathways, while GSA can be further converted into glutamate and into related metabolites.

#### 1.1.3. Kinetic Characteristics of OAT

The basic kinetic properties of OAT have been extensively studied. Some properties are very well conserved among species, as seen in Table 1, which summarizes the known kinetic constants. Of note, available values concern almost exclusively the reaction in the direction of ornithine degradation; only one publication gives *K*_m_ values for GSA (1.4 mM) and Glu (25 mM) for the rat liver enzyme [14]. However, in mammals OAT exhibits very distinct properties depending on the tissue it is isolated from, with major differences between the intestine, kidney and liver. Differences are marked enough to have elicited long debate on whether isozymes exist, but since there is now agreement that there is only one gene for OAT, pseudo-genes not being transcribed or leading to non-functional proteins (see below), other explanations have to be found for these apparent discrepancies.

Several possible explanations will be detailed in what follows, including alternative splicing [29], changes in OAT concentrations leading to different aggregation states with different properties [30], and changes in acetylation and succinylation states.

**Figure 4:**
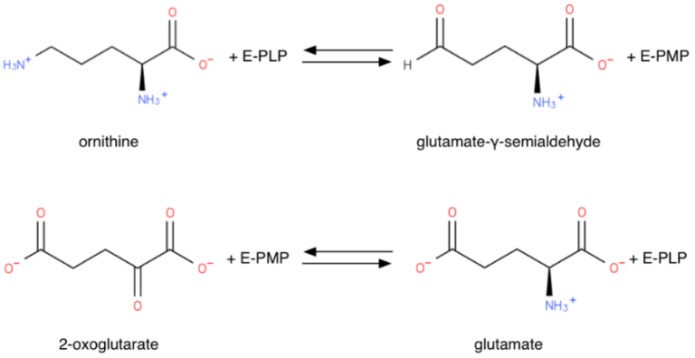
“Ping-pong” mechanism half-reactions of OAT (E-PLP: OAT PLP adduct; E-PMP: OAT PMP adduct).

OAT requires pyridoxal 5-phosphate (PLP) to be functional. OAT, like every PLP-dependent transaminase, operates *via* a “ping-pong” mechanism, two half-reactions completing a full transamination cycle. [12], as shown in Figure 4. Like other transaminases, OAT in the absence of substrates forms an internal aldimine with the PLP cofactor covalently bound on a lysine residue through a Schiff base. In the first half-reaction, ornithine forms an external aldimine with PLP, no longer covalently bound to the enzyme, but retained in the active site through non-covalent interactions. The Orn δ-amino group transfer then transforms PLP into a pyridoxamine phosphate (PMP) intermediate, thus forming a glutamate semialdehyde-PMP aldimine. This intermediate is hydrolyzed to release GSA, leaving the OAT complexed with PMP. In the second half-reaction, aKG forms a Schiff base with the PMP. The amino group is transferred to aKG to form Glu, while the cofactor reverts to its PLP form [31]. OAT thus acts as an ω-transaminase in the first half-reaction, and as an α-transaminase in the second half-reaction: although the α-amino group is more reactive then the distal one, in the first half-reaction OAT transaminates the distal OAT amino group [32]. The structural reason for this ω-transamination selectivity will be explored below in 2.3.

Each half-reaction has its own kinetic profile, with different effects of pH: for the first one, the reaction rate is maximal in the range pH 8–10; for the second one, it peaks at pH 6–8.5 [19]. The enzyme’s optimal pH is thus a compromise: it depends on the organism considered (Table 1), owing to slight variations in the amino acid content of the protein, but is around 8, not far from the mitochondrial pH.

### 1.2. Inhibitors

Besides helping us to understand the mechanisms of action of OAT and its involvement in metabolism, recent advances on the role of OAT in cancer and in other pathological processes (see last part of this paper) have fueled interest in OAT inhibitors [33,34]. OAT is inhibited by aminohexanoate [35], GABA, gabaculine, 5-fluoromethylornithine (5-FMOrn) [20,36,37,38], and l-canaline [39]. A more complete list of inhibitors and their structures is given in Table 2.

An intriguing property of 5-FMOrn is that even at high concentrations, a small OAT activity remains detectable in some tissues (brain and liver), whereas in other tissues inhibition is complete [20]. This activity is associated with kinetic properties that are somewhat different from “standard”; there is yet no satisfactory explanation for this finding. One possibility could be the weak OAT activity of another transaminase: GABA transaminase (EC 2.6.1.19) is a potential candidate, being structurally very close to OAT and present in both brain and liver [34].

## 2. Structure of OAT

### 2.1. Primary Sequence and Leading Peptide

The human OAT (hOAT) sequence is available on Uniprot (code P04181, OAT_HUMAN), along with other OAT or putative OAT sequences (*n* = 53). The “canonical sequence” of the human OAT (Uniprot P04181-1) has a length of 439 AA and a molecular mass of 48,535 Da. A second isomorph is reported in the database (Uniprot P04181-2) with the first 138 residues missing. There has been a long controversy about differences between liver and kidney OAT, some studies suggesting the existence of different isozymes [45,46] and distinct physicochemical properties [41]. However, other researchers have found no evidence for any difference between the liver, kidney and small intestine forms of rat OAT [18,47].

In organisms in which OAT is mitochondrial, the translated protein includes a signal peptide that targets the peptide to mitochondria. In humans, mutations in this peptide are one of the causes of gyrate atrophy.

There is controversy about the length of the leading mitochondrial signal sequence. On UniProt, the cleavage location is reported to change between the kidney (after Q35, based on the work of Kobayashi et al. [48]—unless otherwise specified, residue numbering is for human OAT) and the liver (after A25, based on the work of Simmaco et al. [49]) forms. However, a careful reading of the cited papers is inconclusive; Kobayashi et al. [48] suggest a similar cleavage for the kidney form, and mention a third possible cleavage site, after L32. Cleavage after Q35 was also described by Oyama et al. [50], who found exactly the same sequence between the renal and the liver OAT starting from this residue. A study on human retinoblastoma found a leading sequence of 32 residues, resulting in a mature protein of 407 residues [51]. Another study on rat liver concluded a leader sequence of 34 residues [52]. However, this was contested one year later, and a leader sequence of 25 residues was proposed [49]. A two-step cleavage of the signal peptide may explain why different cleavage sites were found.

The sequence is positive for the Prosite [53] pattern PS00600, characteristic of the aminotransferases class-III PLP attachment site [54]. The consensus pattern is [LIVMFYWCS]-[LIVMFYWCAH]-x-D-[ED]-[IVA]-x(2,3)-[GAT]-[LIVMFAGCYN]-x(0,1)-[RSACLIH]-x-[GSADEHRM]-x(10,16)-[DH]-[LIVMFCAG]-[LIVMFYSTAR]-x(2)-[GSA]-K-x(2,3)-[GSTADNV]-[GSAC], where the conserved lysine residue is the pyridoxal-P attachment site. Among the 1070 UniProtKB/Swiss-Prot sequences having this pattern, 1031 are true positives.

**Figure 5:**
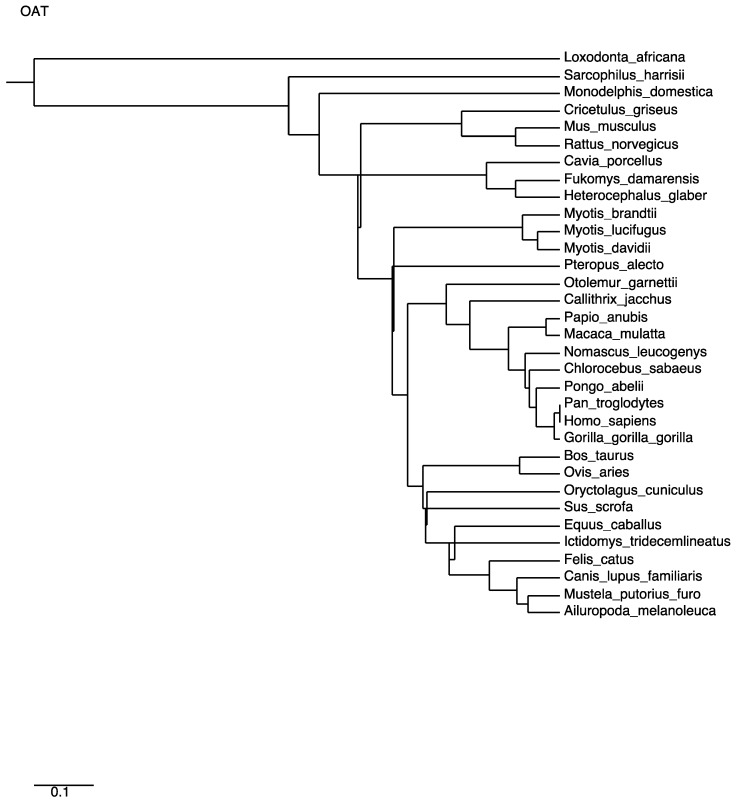
Phylogenetic tree computed from multiple alignment distances of mammalian OAT sequences.

**Figure 6:**
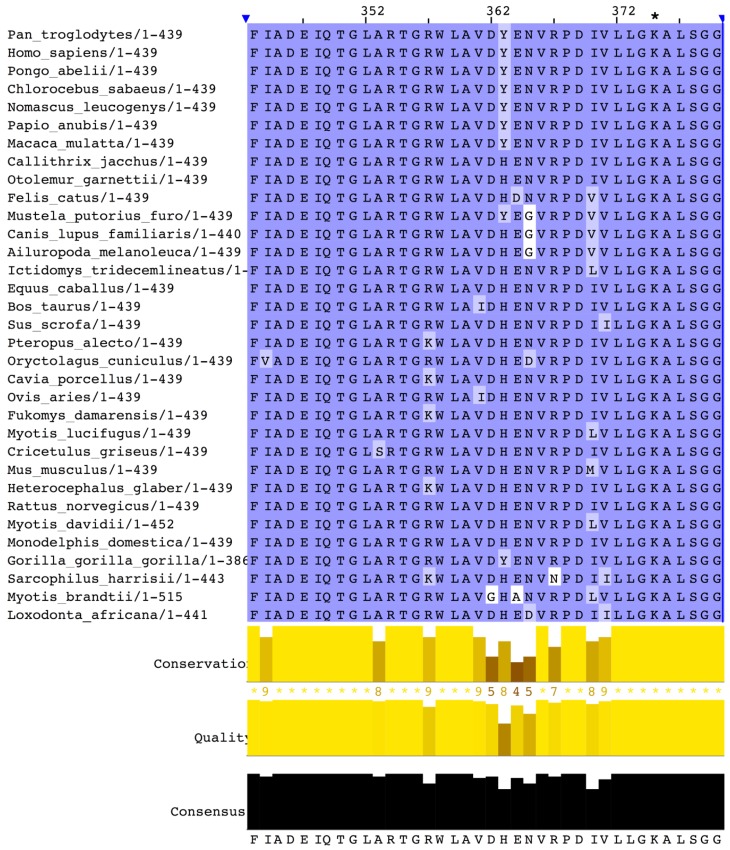
Close-up of the Prosite consensus pattern region of the Clustal multiple alignment of mammalian OAT sequences. The pyridoxal 5-phosphate (PLP) attachment site is the conserved lysine residue in column 375 of the alignment (denoted by an asterisk), which corresponds to residue 292 in the human sequence.

A multiple alignment of mammalian OAT sequences from the Uniprot database yields very high identities. The phylogenetic tree (Figure 5) computed from this alignment is consistent with the close relatedness of all primates (100% identity of human OAT with the common chimpanzee and above 98% for other primates), but also near 90% identity with most mammals (rodents, canids, ruminants or bats), with the exception of elephant sequences, which have 75% identity with the human sequence. A close-up of the consensus Prosite pattern (Figure 6) clearly shows the high degree of conservation of this key region (lysine residue PLP attachment site is in column 375 of the alignment, corresponding to residue 292 in the human OAT sequence).

### 2.2. The Three-Dimensional Structure

OAT belongs to the broad fold-type I structural group of PLP-dependent enzymes, and inside that group to the class-III aminotransferases according to Grishin et al.’s nomenclature [54] (corresponding to the group initially named AT-II by Mehta et al. [55]). Most of these enzymes are ω-aminotransferases, with specific features explained by a number of conserved residues in the active site [56,57]. Six crystal structures of the human OAT enzyme are present in the PDB databank: one of the recombinant enzymes [12], and three enzyme complexes with 5FMOrn [58], l-canaline and gabaguline [59], rerspectively, and two forms mutated on one or three residues [60]. All six structures are isomorphous, and overall structures are almost identical, with only small differences on two flexible surface loops between free and complexed forms or on the mutated residues between wild and mutated ones. Each OAT monomer contains 12 α-helices and 14 β-strands, and forms a large and a small domain. The biologically active form is a dimer formed through extensive hydrophobic and hydrophilic interactions, essentially between the large domains of two monomers, burying approximately 5500 Å^2^ of solvent-accessible surface. The two active sites of the dimeric unit are 25 Å apart. The crystal packing creates a hexamer formed by a trimer of dimers related by an approximately 3-fold screw axis. Although it is not possible to tell from the crystal structure whether the hexamer is of biological relevance or merely an effect of crystal packing, there is experimental evidence for the existence of hexamers of rat liver OAT in solution approaching crystallization conditions [61]. Assemblies of dimers or tetramers have been observed in electron microscopic studies on pig kidney OAT [62], and oligomers are detected in high concentrations of mouse liver OAT [63]. The oligomerization state seems to change the kinetic properties of the enzyme [30], and may partly explain the differences observed between tissues, although the kinetic studies performed up till now have never shown any sign of cooperation between the various subunits (the kinetics seems to be of the Michaelis Menton type, see above).

### 2.3. The Active Site

**Figure 7:**
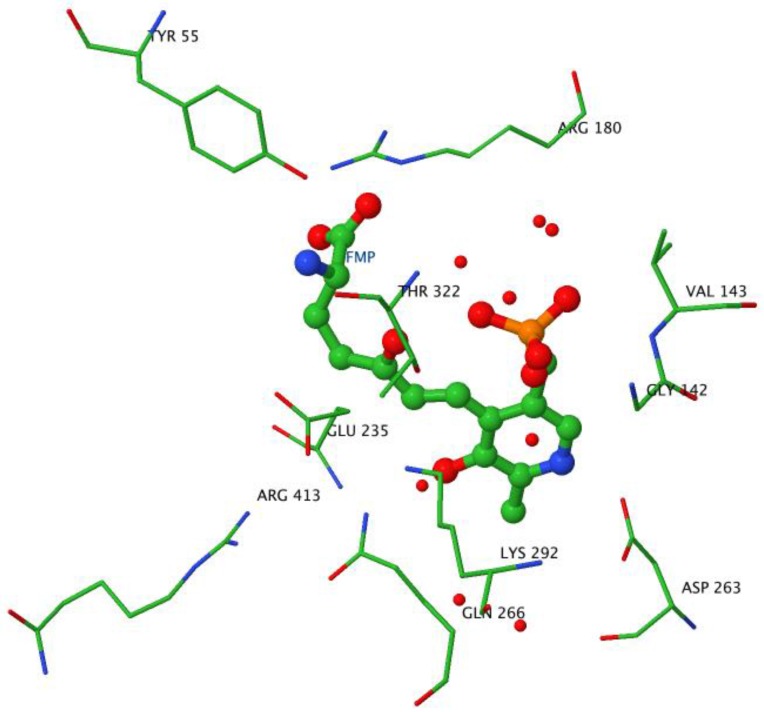
Active site geometry. Image created with JMol using the OAT-5FMOrn crystal structure coordinates. The ball and stick structure shows the adduct formed by the reaction of 5FMOrn with PLP. The wireframe structures indicate the residues stabilizing the adduct in the active site in particular Y55, R180, K292, and the shielding of R413 by E235.

The PLP cofactor is fixed at the interface between subunits in the large domain, forming an internal aldimine via a Schiff base bond with K292. Crystal structures of OAT complexed with inhibitors have been studied in order to elucidate the exact mechanism of the reaction. The suicide inhibitor 5FMOrn is an Orn analogue irreversibly binding in the OAT active site in the initial transamination step [58]. 5FMOrn forms a stable adduct with the PLP cofactor, referred to as FMP, which is detached from the K292 residue, but is kept firmly in the active site through non-covalent interactions with OAT residues (Figure 7). This crystal structure suggests a mechanism for the specific ω-transaminase function of OAT [58]: the α-amino and α-carboxy groups of Orn are anchored to Y55 and R180 respectively (mutations Y55S and R180T on these two residues cause loss of OAT activity, and lead to gyrate atrophy [64,65]); thus only the δ-amino group can approach the reactive Schiff adduct of K292-PLP. Arginine R413 could bind the ornithine α-carboxylate group instead of R180: This arginine (R413) is one of the four residues conserved in all transaminases [55]; in other transaminases, it binds the α-carboxylate group of the substrate through a hydrogen bond [66,67]. Conversely, in OAT, residue E235 forms a tight ion pair with R413, making it unavailable for hydrogen bonding. It is suggested that this shielding leaves no alternative binding residue for the α-carboxylate other than the R180, which accounts for why OAT transaminates the ω instead of the more reactive α-amino group in its first half-reaction. However, this hypothesis was challenged by a crystal structure study of an OAT with glutamate 235 mutated to alanine [60]. This mutation conserved specific transamination of the δ- instead of the α-amino group of ornithine, while at the same time shifting the specificity from ornithine to glutamate. An E235S mutation produced similar qualitative results, but on a lesser scale. In addition, the interaction between residues E235 and R413 is stabilized via a hydrogen bond network involving the PLP form of the cofactor, which is weakened when the cofactor is in its PMP form. The authors [57] suggest that E235 acts as a switch, closed in the E-PLP form, thereby allowing binding of ornithine for ω-transamination, but impeding binding of glutamate, and opened in the E-PMP form, making R413 available to bind aKG. We note that this model is consistent with the experimental evidence that, in vitro, OAT more easily catalyzes Orn degradation.

## 3. Synthesis and Fate of OAT—From Gene to Protein

### 3.1. From Gene to Protein

#### 3.1.1. Genes Encoding OAT

In humans, OAT is coded by a single gene, on chromosome 10, region q26 [68,69] (positions 124397303 to 124418976 in human genome 19; also named OKT, GACR, OATASE and HOGA). In addition, there are at least three pseudogenes of very close sequence similarity: two on the X-chromosome [69] at Xp11.2 [68] (OATL1) and X (OATL2), and one on chromosome 10, next to the original gene (OATL3). Proof of the functionality of the gene on chromosome 10 was established by transfection and expression in hamster ovarian cells [70]. The pattern is similar in the mouse, with a gene on chromosome 7 and pseudo-genes on chromosomes X and 3 [71].

Interestingly, the OAT gene promoter region shares properties of low-level, ubiquitous house-keeping genes (rich GC content, Sp1 binding sites) and binding sites for several transcription factors found for more tissue-specific genes. This finding is consistent with the low levels of OAT found in almost all tissues, and higher expression in a few tissues.

Little is known about regulation by miRNAs, with only one mention in the literature of a putative OAT gene predicted as the target of four different miRNAs in *Amphioxus* [72]. In humans, the OAT transcript is one target of hsa-miR-1 [73,74], which is involved in muscle and heart development. This finding is consistent with the recent discovery of the importance of OAT in muscles, and of its changes during growth [75]. Additionally, the possibility arises of a link between the existence of OAT pseudo-genes on the X chromosome, the fact that pseudo-gene transcripts were recently discovered to act as a trap for miRNAs, and the higher expression of OAT in females. Two teams [76,77] claimed a link between sarcoma cell lines and mutations in the X chromosome near the OATL1 and OATL2 loci. A few other hsa-miRs are also predicted *in silico* to interact with OAT transcript [78], but with no experimental evidence to date.

#### 3.1.2. Transcription

Transcription occurs on the (−) strand, and there seem to be several variants of the transcript. The primary transcript of the OAT gene is 21 kb long and contains 11 exons. Exon 2 is, however, spliced out [79], except in some retinoblastoma cell lines, where a few mRNAs escape this splicing [80]. Except for this particular case, there is no experimental evidence for regulation through alternative splicing.

Transcription control by glucagon and glucocorticoids of the urea cycle enzymes, as occurs in the liver, needs a CCAAT regulation site and the fixation of the CCAAT enhancer-binding protein β [81]. Since the OAT gene promoter also has CCAAT regions, and glucagon also increased OAT activity in the liver, a similar mechanism is probably in play.

#### 3.1.3. Translation

Translation starts in exon 3, and seems to occur on free polysomes [82]. It involves the eIF-4E translation factor [29], except for the few mRNAs that do not splice out the exon 2, which are less translated, and independently of the eIF-4E level. The reason for this is not clear, two possible explanations being the fixation of a protein (or of miRNA) and the longer prefix sequence, facilitating the formation of mRNA secondary structures inhibiting the binding of translation factors. Protein-protein interaction studies have also identified eIF-6 as interacting with OAT [83], suggesting it may also be involved.

### 3.2. Changes in Protein

#### 3.2.1. Peptide Maturation

Since OAT is mitochondrial in most species, the translated peptide must be translocated into the mitochondrial matrix. This is done using a signal peptide that is removed after translocation. Orientation toward the mitochondrion first occurs by the binding of the precursor to a receptor on the mitochondrial outer membrane; the signal peptide is removed after crossing this membrane [84]. As dealt with in detail in 2.1, there is some controversy about the exact length of this signal peptide.

#### 3.2.2. Post-Translational Modifications

**Figure 8:**
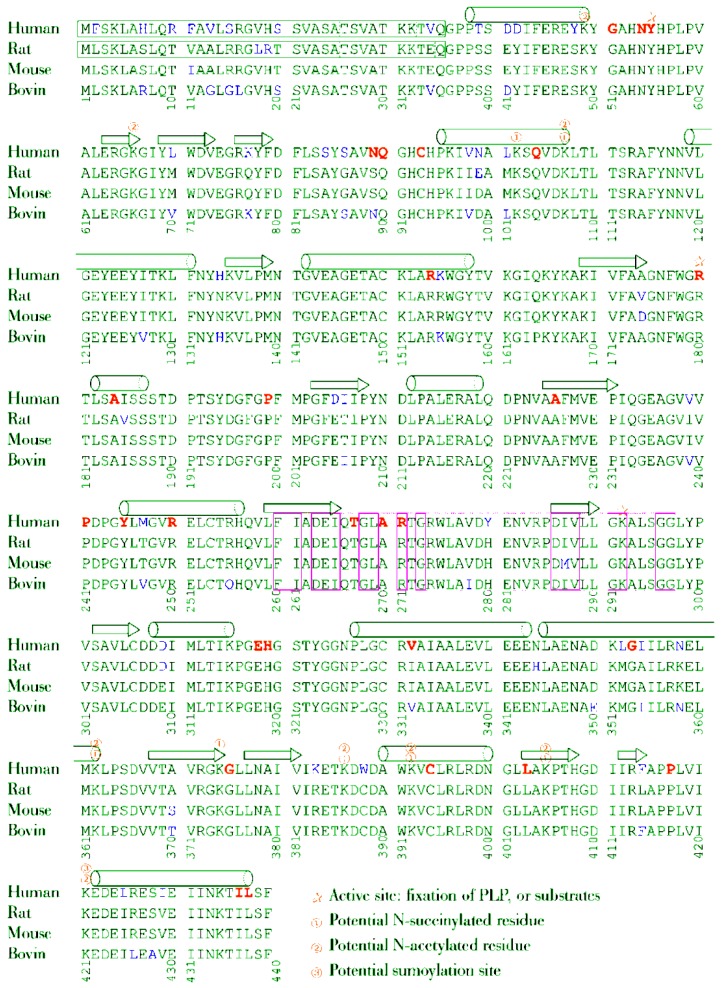
Amino acid sequence alignment of human, rat, mouse and ox OAT.

Three kinds of post-translational modifications are experimentally proven for mammalian OAT and are summarized in Figure 8. The first one is the fixation of the PLP cofactor in the active site, already described in detail (2.3). The second one was detected in mice, and corresponds to *N*-acetylation and *N*-succinylation of lysine residues. Seven Lys residues of OAT can be succinylated in mouse liver [85] in positions 102, 107, 362, 374, 386, 392 and 405, and desuccinylation occurs through Sirt-5, a sirtuin enzyme. Conversely, 8 Lys residues can be acetylated [86] in positions 49, 66, 107, 362, 386, 392, 405 and 421, and deacetylation occurs through Sirt-3. We note that five of these positions are common to succinylation sites. The exact consequences of these post-translational modifications are not known. They may be involved in the differences in OAT kinetics noted among various organs (see section 1).

There seems to have been no experimental mention to date of any other post-translational changes, including a potential enzyme regulatory modification (e.g., nitrosylation, phosphorylation). However, large-scale experiments and computer predictions suggest that sumoylation [87,88], neddylation [88,89] and ubiquitination [90,91,92,93,94] can occur, and that there may be several kinase target sequences.

### 3.3. Life and Death of the Protein

#### 3.3.1. Interaction with Other Proteins

Several protein-protein interactions involving OAT have been predicted *in silico* or found in large-scale studies, prompting several hypotheses on the functions of OAT and on its regulation. Proteins involved, detected in a very large-scale co-precipitation study [95], with some of the interactions that were only predicted, are NME2 (nucleotide diphosphate kinase, involved in tumorigenesis), EZR (ezrin), the aspartate aminotransferase GOT1 (or more probably GOT2, its mitochondrial equivalent); ESD (esterase D, a marker of retinoblastoma), AKR1B1 (reducing aldehydes and ketones), ALDH1B1, CBS, SOD1 and SOD2, PDXK (pyridoxal kinase), UAP1L1, UQCRFS1, PREP, PGM1, PITPNB, PPID and ADSS, the biological relevance of which is still difficult to assess. Other studies have detected interactions with NTRK1 [96], HDAC5 (a histone deacetylase [97]), FAF2 [98], NUDT3 [99], SIRT-7 [100], ICT1 [101], SLC35F6 [102], OTUD4 [103], DMWD [103], CDK2 [104], UBQLN4 [105], HUWE1 [106], FUS1 [107], EGFR [108], ADRB2 [109], FBXO6 [110], CTSA [111], ATF2 [112]. However, to our knowledge, there is no experimental confirmation that these interactions occur in normal cells under physiological conditions. For instance, interaction between ATF2 and OAT was detected in an experiment in which it was concluded that ATF2 targeted the mitochondrial outer membrane through interactions with its proteins, but since OAT is in the mitochondrial matrix, the reason for the detection of ATF2-OAT pairs is not clear.

A special case is the interaction with c-MYC, a transcription factor involved in cell proliferation: protein-protein interaction was detected in a large-scale study [113], but conversely, c-MYC was found to increase the OAT mRNA levels, and other enzymes involved in Glu metabolism, in activated T-cells [114], which use Glu as a precursor for Orn, which in turn is a precursor of polyamines. Hence this OAT-cMYC interaction might be a negative feedback, but no experimental evidence has yet provided any insight into the precise role of this interaction.

#### 3.3.2. OAT Degradation

The OAT degradation rate constant is around 0.4 day^−1^ in the liver [35,115,116], i.e. a half-life of about 1.7 days (but only 0.95 days in the rat [117]). The degradation rate seems to be much slower in the kidney, with a half-life of about 4 days in the rat [117]. It may start by the loss of its PLP cofactor [118].

Since sumoylation, neddylation and ubiquitination are usually involved in protein degradation control, the occurrence of these various binding sites may explain differences in OAT half-lives according to the different organs.

## 4. Localization of OAT

### 4.1. Cellular Localization

In all known species with OAT activity, OAT is a soluble, intracellular protein.

In eukaryotic cells, the cellular localization of OAT varies among different phyla. It is a cytosolic enzyme in fungi (shown for *Neurospora* [119] and *Saccharomyces cerevisiae* [120], and suspected for other fungi (*Agaricus bisporus* [51,121], *Saccharomycetae* [26,121,122])) and in most unicellular organisms, but it is mitochondrial in most plants and vertebrates. In all mammals investigated thus far OAT is in the mitochondrial matrix. This localization is consistent with the fate and origin of the metabolites it uses, since most pathways with which OAT interacts involve mitochondrial enzymes (Figure 1). This mitochondrial localization may also control the reaction kinetics, OAT activity being apparently limited by the rate of ornithine entry into the mitochondrion [22].

There are also consistent reports that OAT is present in the cell nucleus, where its function is still unknown. Several observations suggest it could be an actor in cell division and function. First, inhibition by diazonamide A, a natural toxin with anti-mitotic properties, was shown to act through OAT in cancer cell lines [123], but without inhibiting its activity. Surprisingly, this interaction does not seem to block normal cell division, but only tumoral cell division. Second, a recent investigation of the changes in the Staufen-1 complex induced by HIV identified OAT as one of the numerous proteins involved in this complex, which binds mRNAs and targets them to different cellular organelles (of note, OAT mRNA was not identified as one of the mRNAs bound to various Staufen complexes) [124]. This observation may be related to the finding that mutations in mitochondrial enzymes, such as OAT, influence HIV-1 propagation [125]. Third, bioinformatics analysis of the OAT gene sequence predicts the existence of binding sites for several transcription factors involved in cell cycle or cancer genesis, such as EVI-1 (two sites). A direct effect of the c-MYC transcription factor was also demonstrated [114]. However, to our knowledge, no experimental evidence for the binding of these transcription factors to the OAT gene has been reported in the literature. Hence future investigation of the nuclear function of OAT may reveal unexpected functions.

### 4.2. Tissue Localization

Before summarizing the information about OAT location in mammalian organs and tissues, some discussion of the experimental methods used are needed, to reconcile apparently conflicting results. Historically, OAT has been first detected in organs and tissues through its catalytic activity, monitored in general by the detection of ^14^C-radiolabeled Orn degradation products, either glutamate or P5C, or by chemical reactions of the product. This has been done on enzymes of varying purity, organ extracts or even in vivo. These experiments, especially if the enzyme was purified, yield conclusive evidence that the enzyme occurs in an active form in the organ considered. However, it gives no insights into its exact localization in the tissue, and seldom into the exact reaction taking place physiologically, because the reaction direction is determined by the relative concentrations of substrates and product.

**Figure 9:**
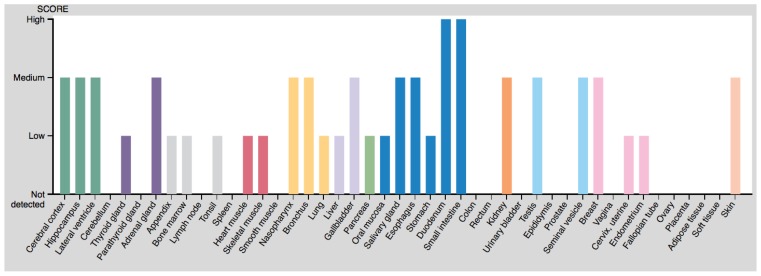
Protein expression scores from the Human Protein Atlas.

Later, direct detection of the protein was achieved using specific antibodies. Coupled with fluorescence, this allows the precise detection of the protein in each cell, thereby giving precise information on the histological location of OAT. However, failure to detect the protein may also mean that it is in a different conformational state or has undergone different post-translational modifications, not recognized by the antibody. It may also result from the antibody binding site being masked by an interaction with another protein. In addition, it tells us nothing about the activity of the enzyme. Typical histological views of OAT localization can be found in the Human Protein Atlas [126,127], obtained using two different antibodies (Figure 9).

**Figure 10:**
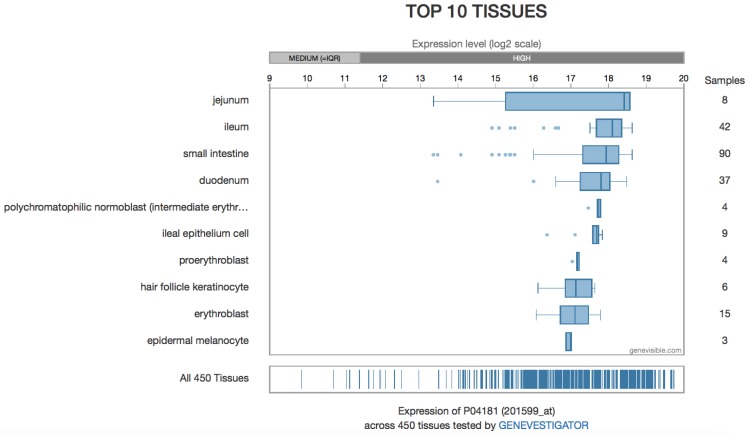
Protein expression levels from microarray data.

The most recent detection method is the semi-quantitative detection of OAT mRNA, as can be found in several gene expression databases, such as the Human Protein Atlas or Bgee [128,129] (although older experiments also quantified mRNA in some circumstances and development of micro-arrays greatly scaled up these data). However, there is not necessarily any correlation between the amount of mRNA and the protein content or its enzymatic activity. Expression levels based on microarray data are available [130,131] (Figure 10).

OAT can be found in almost all tissues (see Table 3), but its activity predominates in the liver, kidney, intestine, and retina. As regards protein concentration or mRNA content, the intestine is clearly the organ in which OAT is most abundant, with both high mRNA and high protein content, whereas other tissues have moderate protein content and low RNA levels [Protein Atlas.org]. Discrepancies between activity and protein content measured by immunochemistry may result from different aggregation of the protein when its concentration changes (see 2.2), with effects on both its activity [30] and the antibody fixation.

### 4.3. OAT in the Liver

As noted above, the liver is one of the organs with the highest OAT activity, and one of those most widely studied in the mouse, rat and pig, and in humans. Interestingly, despite this high activity, RNA levels and protein levels detected by immunohistochemistry are quite low, except in the gallbladder [136]. Beside the explanations given above, another possibility is the change in cell conditions, liver OAT activity depending on the protein content of the diet.

Whereas early work suggested OAT involvement in ornithine synthesis [46,137,138,139] to modulate its availability for the urea cycle, further experiments suggested that OAT instead removed excess ornithine to prevent an increase in urea cycle metabolites [22]. Involvement in gluconeogenesis has also been proposed [140].

However, most of these experiments were conducted using all types of hepatocytes (i.e., periportal and perivenous), complete liver, or even in vivo: thus, the results must be interpreted with caution because expression experiments suggest that OAT and the urea cycle enzymes are expressed in distinct cells [141], with OAT expressed in pericentral hepatocytes in the adult mouse [142] and rat [143]. This is in line with histochemical experiments carried out on rats [144], which found an increasing gradient of OAT from the periphery to the center of the lobule. Colocalization of OAT and glutamine (Gln) synthase in these cells suggests that OAT might be used to generate Glu: With the rapid reduction of GSA to Glu in the mitochondria [22], each ornithine molecule allows the generation of two Glu through this pathway [139]. Since aKG, as a Krebs cycle intermediate, should not be limiting, this seems to be a very efficient way to supply Gln [145]. Interpretation of OAT activity experiments in the liver, without considering cell type and localization in the lobule, may also be biased by a potentially higher expression of OAT in the gallbladder [136], and so putatively in its afferent canals. The role of OAT in the gallbladder is unknown, but the OAT gene promoter may contain an aryl hydrocarbon receptor (AhR) and an AhR nuclear translocator (ARNT) binding site [*in silico* prediction], and AhR is known to bind bilirubin; this opens a possible line of enquiry.

In the liver, OAT activity increases after a high protein diet [146,147,148], an amino acid-enriched diet, or stimulation by glucagon [149,150] or cAMP [140]. Effect of amino acids is indirect and mediated by glucagon, which acts by increasing cAMP levels. However, this action occurs only in the presence of (or after a pre-treatment by) cortisone [150]. Glucose, fructose, l-sorbose, and glycerol seem to inhibit this regulation process [140,146,148,151], with an efficacy that varies between in vivo and cultured cells. In all situations, the effect seems to be on the translation step, with increased rate of synthesis [149], and little or no effect on transcription levels [152]. How hexoses and glycerol act is not yet fully understood. OAT activity is also increased by all-*trans*-retinoic acid (a*t*RA) [153], through transcription enhancement.

Conversely, OAT activity is decreased by glucocorticoids [154,155], and by lipopolysaccharide (LPS) [156]. In mice and rats, it presents circadian variations, with a decrease along the day, by control of the translation step, but with no change in the mRNA levels [17,149,157], and with an effect of the dark/light transitions [158]. Oxygen pressure also seems to have an influence, with low levels increasing OAT activity, which may be responsible for the higher activity of OAT in pericentral hepatocytes [143]. The mechanism is not completely elucidated, but may involve a heme protein and share the same mechanism as glucagon and cAMP. Another explanation could be the increased NH_4_^+^ concentration at low O_2_ pressure (owing to a slower urea cycle rate), lowering OAT degradation. A third, putative explanation based on *in silico* predicted features of the OAT gene is the presence of an ARNT binding site, ARNT being known to interact with HIF-1a to form hypoxia-induced factor 1 (HIF1). Since the experiments of Colombatto et al. [143] did not directly show the involvement of a heme protein, but only that cobalt chloride increased OAT activity (even though CoCl_2_ is a known direct inhibitor of OAT [23]), and since it is known that CoCl_2_ induces HIF-1 [159], this regulation pathway seems consistent, although it awaits confirmation.

### 4.4. OAT in the Digestive Tract

In the intestine, OAT is believed to catalyze the formation of Orn, which is subsequently converted into Cit, and ultimately into Arg under conditions where argininosuccinate synthase (ASS) and argininosuccinate lyase (ASL) are expressed (see below for details). The intestine is the main organ presenting this flux characteristic. The importance of this reaction changes during growth, as will be detailed in 6.1 (briefly, in neonates Pro is an Arg precursor, and lack of OAT has adverse effects; mice lacking OAT are not even viable without Arg supplementation in the first weeks); the focus here will be on adult mammals, in which OAT is used to produce Cit from Glu. Considering the equilibrium constant of the reaction, taking this direction requires either a large amount of GSA and Glu, or fast consumption of ornithine (or aKG). This is in line with its conversion into citrulline, which in turn is released in the portal circulation. Large amounts of Pro may also lead to ornithine synthesis by OAT [160].

In the small intestine, none of the activators at work in the liver or kidney seem to have any effect. It has been shown that a low protein diet induces intestinal OAT [161], and that all-*trans*-retinoic acid (a*t*RA) induces an increase in OAT activity during the differentiation of villus cells [162].

### 4.5. OAT in the Kidney

In the kidney, OAT converts ornithine and α-ketoglutarate into P5C and Glu, respectively. Interestingly, females have about twice as much OAT protein as males [18]. In mice, OAT activity seems to be higher in the proximal than in the distal tubule [163].

In the kidneys, OAT activity is insensitive to glucagon and high protein diet [164], but is increased by thyroid hormones [165] and estrogens [117,152,164,166]. By contrast, testosterone seems to decrease its activity [167]. This stimulation by estrogens and inhibition by testosterone doubtlessly explains the higher OAT activity in female kidneys. Tri-iodotyrosine (T_3_) may act through cAMP. Estrogens seem to act by accelerating the translation initiation, whereas T_3_ changes the transcription [168].

### 4.6. OAT in the Brain and the Nervous and Sensory System

In the brain, OAT is found in cortical, hippocampal and basal ganglionic neurons [169] and, at a lower level, in glial cells [136]. It is thought to be used for Pro synthesis [135], but has also been proposed as a source of Glu, a precursor for different kinds of neurotransmitters (Glu itself [170], GABA [171,172,173]). This last idea is supported by the high amount of OAT found in nerve endings [174], and by a decrease in OAT activity in brain anoxia models, in tissues where GABAergic neuron degradation occurs. However, other experiments suggest that GABA is not derived from Orn [42,170]. GABA itself has an inhibitory effect on OAT that could be due to substrate competition with Orn (see above) [42]. This was observed in vivo by peritoneal administration of vigabatrin, leading to an increase in GABA levels and Orn levels, and confirms the Orn → GSA/Glu function of OAT in the brain.

The link between OAT and Glu as a neurotransmitter has been mentioned to account for the lower levels of OAT found in the brains of patients who have died from Huntington’s chorea, compared with other dementias [175,176]. Another putative connection is the well-established fact that bilirubin is toxic for nerves, and that its toxicity is accompanied by increased Glu: since the OAT gene may have an AhR binding site (see above) and AhR binds bilirubin, exploring OAT expression and activity may help to elucidate the mechanism of bilirubin toxicity.

OAT is also found in several parts of the eye (Table 1; [132,177,178]). Although its role is currently unknown, this finding is consistent with the ophthalmic consequences of OAT dysfunctions, as seen in gyrate atrophy (see 6.2). OAT activity in human retinoblastoma cells seems to be regulated as in kidney cells [80].

### 4.7. OAT in the Other Tissues

In other organs and tissues, OAT is viewed as a housekeeping protein, controlling ornithine availability for the cell: microarray expression experiments have detected OAT mRNA in at least 131 different types of tissue [Bgee gene expression database] [129]. As Orn is a precursor for polyamines, OAT is involved in controlling the proliferation of several cell lines, such as colonocytes [179] and vascular smooth muscle cells [180]. There is also evidence that OAT is involved in activated T-cells, to provide both ornithine and aKG [114]. Conversely, controlling the metabolism of Orn into Pro makes OAT a modulator of collagen synthesis, and thus of extracellular matrix formation [180].

Attention has recently focused on OAT in muscles [181]. In terms of the protein atlas, the protein was found in heart and skeletal muscle cells, but not in smooth muscle cells, whereas RNA is found in all kinds of muscle cells. Often underestimated, the role of muscle OAT seems particularly important: although specific OAT activity per unit mass of protein in muscle is lower than in other tissues (intestine, liver and kidney), the much higher muscle mass in the body compared with these organs, implies a muscle contribution to whole body OAT activity similar to that of the liver in adult mice [75]. The exact role of OAT in muscle is unclear. The OAT regulation in the muscle seems similar to that in the kidney, with lower levels in males than in females, which may result from either estrogen stimulation in women or testosterone inhibition in men.

## 5. The Role of OAT in Ornithine Fluxes

As noted above, the reaction catalyzed by OAT favors Orn degradation in vitro, but there is evidence that the equilibrium constant is low enough to allow Orn synthesis under certain circumstances in vivo. The reaction direction is controlled by relative metabolite concentrations, which is difficult to appraise without a general view of the pathways in which OAT is involved. Throughout the following discussion on Orn fluxes we must keep in mind the direction of flux to be considered: net Orn degradation by OAT at the whole body level does not preclude net Orn synthesis locally in some specific organs or in particular situations.

### 5.1. Fluxes in Non-Mammals: Orn Degradation

For organisms other than mammals, the picture seems quite simple: all the available evidence suggests that OAT catalyzes Orn degradation into Glu and GSA/P5C. The end-product of ornithine by this pathway can be either Glu or Pro, and there is frequent controversy about which it is. This simplicity may be related to the existence of a completely separate pathway producing Arg, involving acetylated derivatives of amino acids.

#### 5.1.1. Prokaryotes

In prokaryotes, OAT (*rocD* gene) is involved in one of the Arg degradation pathways, allowing the formation of Pro, the first step being Arg deimination into Cit, which in turn is further converted into Orn. However, the full picture may be more complex, because the arginine biosynthesis pathway involves a closely related enzyme, *N*-acetylornithine δ-aminotransferase (EC 2.6.1.11; NAcOAT, *argD* gene), which can also act on ornithine. Furthermore, this degradation pathway is not the main one, and is used in conditions where no better source of nitrogen than Arg is available. Quite often only the first two steps take place, as an energy-producing pathway. Lastly, arginine catabolism can also proceed through succinylated compounds; an *N*-succinylornithine δ-aminotransferase (EC 2.6.1.81, SOAT) operates, is close to OAT, and may also act on ornithine. Hence proving the existence of a functional OAT, distinct from NAcOAT and SOAT, is difficult: its existence in Archaeobacteria is, for example, still debated, as to date it has been detected only in genome analysis. Even the existence of the gene in the *roc* operon is no proof, recent work having shown that *Mycobacterium tuberculosis* (and other species causing tuberculosis) has a non-functional *rocD* gene, unlike other *Mycobacteria* [182].

#### 5.1.2. Plants

In plants, where the arginine biosynthesis pathway also uses *N*-acetylated precursors in the production of ornithine, the situation is similar to that in bacteria, and OAT is believed only to degrade ornithine. However, its role is a subject of debate and may depend on the organism. Some authors claim that OAT is involved in resistance against some kinds of stress, especially salt stress and drought stress, through the synthesis of Pro (which is regarded as a reactive oxygen species [ROS] acceptor and as an osmolyte) that accumulates in vacuoles. OAT activity is indeed increased by salt (NaCl) stress in radish seeds (*Raphanus sativus*) [121], probably through increased translation from already-formed mRNA [183], *Arabidopsis thaliana* seeds [122], and by drought and oxidative stress in rice (*Oryza sativa*), where OAT gene transcription is under the control of an *SNAC2* gene that codes for a transcription factor involved in the stress response [184]. However, in other plants such as *Vigna aconitifolia*, this pathway is used when nitrogen is in excess [28], and other works suggest that OAT is used in the seeding process to enhance Gln production. Inclusion of the *A. thaliana* OAT gene in other plants induced an increased resistance to drought and salt stress that may be useful in their cultivation (e.g., tobacco, *Nicotiana plumbaginifolia*, or squash, *Cucurbita pepo*) [121].

#### 5.1.3. Fungi

In fungi, from which one of the first OATs was identified (in *Neurospora crassa* [185]), OAT seems to be involved in the synthesis of Pro [186] (*Aspergillus nidulans*) and Glu.

#### 5.1.4. Unicellular Eukaryotes

In unicellular eukaryotes, OAT also mainly acts as an ornithine metabolizing enzyme. It can be a step towards Pro production (as in *Acanthamoeba castellanii* [187], but it is also seen as a way to eliminate Orn in excess. Since Orn is the precursor of polyamines, involved in cell division, OAT is an actor in cell division control. This role was demonstrated in particular in *Plasmodium falciparum*, the agent of malaria, and probably in other *Plasmodia* (but not all; the OAT gene seems to be truncated in some of them) [26,188].

### 5.2. Fluxes in Mammals: A Subtle Compromise between Orn Degradation and Orn Synthesis

The pattern in mammals is far more complex, probably because the specific pathway for Arg synthesis through *N*-acetylated metabolites is not functional. The same enzymes, including OAT, are thus used in both directions.

#### 5.2.1. OAT and Orn Degradation

As can be inferred from observations in other organisms, and for the various reasons given above, the main net flux (whole body) is from Orn to GSA/P5C [6,22,134,189,190,191]. This is also illustrated by the hyperornithinemia observed in OAT-deficient (gyrate atrophy) patients [192,193,194]. Conversely, OAT overexpression in transgenic mice decreases plasma and liver ornithine concentrations [195], also consistent with the ornithine catabolism direction.

Some indications at the organ level of the directionality of the OAT reaction have already been given in 4.2. Briefly, in most tissues, OAT catalyzes Orn degradation. In the liver, this is a way to generate Glu for Gln synthesis and eliminate excess nitrogen, consistent with its induction by amino acids and high protein diets and its localization with Gln synthase (see 4.2). In the brain, generated Glu is a precursor for neurotransmitters. In general, in other tissues and at the whole body level, OAT is thought to remove excess Orn to avert its toxic effects.

#### 5.2.2. OAT and Orn Synthesis

Intestinal OAT is clearly involved in Orn synthesis to generate Arg or aliphatic polyamines. Several studies show that OAT does not work in this way in the liver, allowing the conversion of excess Orn (generated from Arg in the urea cycle) into P5C [22]. However, it is noteworthy that in the small intestine, OAT works in the reverse direction (P5C → Orn) [196], likely due to its high OAT and low PCDH content [1]. Another study supports the hypothesis that ATP and NADPH can be used to reduce Glu to glutamate-γ-semi-aldehyde, which is subsequently aminated by OAT to yield Orn in the small intestine [197,198].

The purpose of Orn synthesis by intestinal OAT differs according to the situation: to generate Arg for growth and development in early infancy, or metabolic adaptation to a fasting state (see below). Changes in tissue-specific OAT activity during growth are observed, and will be detailed below in 6.1. Briefly, in neonates, the gut generates Arg from Pro (found in maternal milk), the kidney and liver not yet being fully functional. In adults, the metabolic process is more complex: in the fasting state, or after a low protein diet/Arg-deprived diet, amino acids must be spared, and the gut produces Cit from Gln [199]. Cit is released in the portal vein and bypasses the liver, without activating the nitrogen elimination pathways (urea cycle and Gln synthesis). This is consistent with the disappearance of ASS and ASL activity in the adult gut [200]. Cit, released in the portal vein, is in turn taken up by the kidney and converted into Arg [201]. OAT plays a key role in this metabolic pathway, at the crossroads where all these sub-paths meet (Figure 1), and simulation studies suggest that it may be the enzyme that controls the kinetics of the Gln to Cit conversion [202], in agreement with its up-regulation in low-protein diets. Evidence that Gln is a precursor of Arg and Cit has been reported for mice [199].

However, the fate of Orn synthesized by OAT could also be in the synthesis of polyamines, through Orn production. Experiments with ^13^C-labeled Gln in activated T-cells has indeed shown the appearance of labeled Orn, accounting for 30% of total Orn [114], but this pathway seems absent in resting T-cells [114]. This was also demonstrated in the proliferation of some types of tumor, by rendering the tumor independent of an external Gln supply. In both cases, OAT is a potential target for new treatments, and a patent has been registered [33].

Of note, this “reverse” pathway seems to exist only in addition to the “catabolic” one, and to date there is no example of an organism that uses OAT solely as an Orn synthesis enzyme. It therefore seems to be an evolutionary acquisition for organisms that lack a specific Arg biosynthesis pathway; it may be related to the need for discrete feeding, and so more flexibility in amino acid supply and storage, associated with pluricellular, mobile organisms like Animals.

## 6. OAT in Health and Disease

### 6.1. OAT and the Development of Mammals

#### 6.1.1. OAT and Arg Supply in the Pre-Weaning Period

As mentioned in 4.1, intestinal OAT plays a major role during development, as Arg synthesis requirements change from the fetus through weaning to adulthood. Conversion of Pro from maternal milk into Arg has been shown to be considerable in pig [203] and mouse [204] neonates, and there is evidence that human neonates also convert Pro into Arg by this pathway [205,206]. The OAT requirement seems to be all the greater as growth is fast, and newborn mice with non-functional OAT die in a few days without an Arg supply. Milk in mammals is poor in Arg, and high net Arg production by the gut is required to sustain growth [207]. Surprisingly, in contrast to what is observed in mice neonates, loss of OAT activity in human newborns (although it leads to seizures due to hyperammonemia, and slower growth) is non-lethal. This may be because of the faster relative growth rate observed in mice neonates, implying Arg requirements exceeding the availability of Arg in maternal milk [204].

The reaction can proceed in this direction because there is a large amount of GSA (in agreement with the high Pro content of maternal milk), and rapid consumption of Orn, which is converted into Cit. In addition, enterocytes in newborns exhibit high ASS + ASL activities, allowing Cit conversion into Arg [208,209]. We can suggest that Orn production is high enough in neonates compared with Orn catabolism for the whole body flux of the OAT-catalyzed reaction to be reversed, with a net Orn synthesis.

#### 6.1.2. Changes in Tissue-Specific OAT Expression

##### 6.1.2.1. Pre-Weaning Period

The pre-weaning period in rats is characterized by a high intestinal OAT activity (higher than that observed in any other rat tissue) [189]. As shown for pigs and rodents, in human neonates, OAT intestinal activity is at its maximum at birth [206], and then declines after three years to reach the adult level at five years [206].

In mice, we note an increase in liver and muscle OAT activity parallel to corticosterone levels. Thyroid hormone may also be involved, and could positively regulate OAT expression [75]. It can be postulated that during pre-weaning, a period characterized by restricted Arg supply (see above), the flux in muscle OAT reactions takes the direction of Orn synthesis in order to spare Arg [75].

##### 6.1.2.2. Weaning Period

Weaning is associated with a reduced intestinal OAT activity [204]. It is noteworthy that weaning by itself leads to a marked decrease in muscle OAT (as observed in early weaning) [75]. It can be suggested that muscle OAT activity is modulated by the transition from a milk diet (rich in lipids and relatively poor in proteins) to a diet rich in proteins [75]. Although a very high protein enrichment (up to 70 % of diet) is known to upregulate liver OAT activity, the impact of this weaning-associated dietary change on liver OAT activity is not clearly established [75,161].

##### 6.1.2.3. Post-Weaning Period and Puberty

In the post-weaning period, the onset of sex hormone secretion leads to several tissue-specific changes in OAT activity. Testosterone secretion in male mice is associated with an increase in OAT activity in the liver and to a decrease in skeletal muscle [75] and kidney [167]. The role of testosterone in the ontogenic profile in male mice is also supported in particular by the impact of orchidectomy on OAT activity in liver and muscle [75]. In rats, 17β-estradiol upregulates OAT activity in the kidney [137,164]. The impact of sex hormones on tissue-specific OAT activity could explain the marked sexual dimorphism observed.

These results suggest a tissue-specific regulation of OAT activity during development.

These specific features imply a fine regulation of OAT activity during development. This has been confirmed experimentally [162,189,210,211,212,213], and may occur through a*t*RA, which increases OAT activity in Caco-2 cells through increased mRNA expression [214]. Interestingly, the presence of intestinal OAT during fetal development in pigs suggests the importance of Orn, Arg, Pro and polyamine synthesis for the development of the intestine during gestation [162]. In neonatal mice, intestinal Orn is probably not used for polyamine production [215]. Of note, several transcription factors involved in development are predicted to have binding sites in the OAT gene promoter (including PAX-4, NKX5-1, HNF1, HNF1a, En1).

#### 6.1.3. Other Implications

OAT plays an important role in Arg synthesis, but there is some evidence that OAT is involved in other aspects of development—possibly through its control of Orn availability for polyamine synthesis, such as in villous cell differentiation (see 4.7, OAT in the other tissues).

During the embryo phase, a relatively high, stable level of OAT is found in the pigment epithelium of chick embryos, suggesting a significant early role of OAT in the maintenance of pigment epithelium function. Surprisingly, a decrease in OAT activity during the same period is observed in the retina [AG27]. These results suggest that OAT plays a particular role in maintaining pigment epithelium and retinal function, as confirmed by the adverse impact on ocular function induced by loss of OAT activity (see 6.2). Lastly, brain regional distribution of OAT activity showed that OAT may be concentrated in glutamatergic neurons, suggesting a role of OAT for the synthesis of neurotransmitter Glu [175]. Studies in *Xenopus laevis* also suggest a crucial role of OAT in the control of neurogenesis during development [216].

### 6.2. Consequences of OAT Deficiency: Gyrate Atrophy of the Choroid and Retina (GA)

OAT deficiency is the cause of a rare, serious and disabling congenital metabolic disorder characterized by gyrate atrophy (GA) of the choroid and retina [194]. Some details on causal mutations, and their consequences on the enzyme structure or activity, can be found in section 2; other mutations on non-coding regions of the gene are presented in section 3.

#### 6.2.1. Overview

As stated above, OAT deficiency in humans results in GA. To date, 65 OAT mutations have been identified according to the Human Gene Mutation Database [217]. This rare congenital metabolic disease is found worldwide, with a particularly high incidence in Finland, where its frequency is approximately 1/50,000 [218]. GA is an autosomal recessive disease clinically characterized by a childhood-onset visual disorder with myopia, followed by the development of night blindness, cataracts and progressive constriction of vision fields, leading to blindness in the fourth to fifth decade [219]. Although in the large majority of cases cognition is unimpaired, a few patients have been described with mental retardation [220]. Biologically, patients with GA display an increase in plasma Orn concentration (10–20-fold) [194], and in some cases present an overflow ornithinuria, lysinuria and cystinuria when plasma Orn exceeds 400 µmol/L [221,222]. It is noteworthy that the increase in plasma Orn occurs after the first 3 months of life [204]; the importance of this metabolic feature is underlined below.

#### 6.2.2. Pathophysiology

Although the cause of GA is well-identified, the physiopathology leading to chorioretinal degeneration is not fully understood. Current assumptions implicate the toxic effect of elevated intraocular concentrations of Orn and its metabolites in excess, such as spermine, on the retinal pigment epithelial cells [223], together with Pro deficiency in the choroid and retina [224].

The cause of mental retardation is unclear and a subject of debate. One likely hypothesis is brain creatine (Cre) deficiency secondary to Orn accumulation: high Orn concentration inhibits arginine-glycine amidinotransferase (AGAT), which results in deficient Cre synthesis [225]. It has been shown that Cre deficiency causes severe neurologic symptoms as observed in cases of guanidinoacetate methyltransferase (GAMT) deficiency. In cases of GAMT deficiency, a high-dose Cre therapy (4–8 g/day Cre monohydrate) nearly normalizes biochemical and MRS parameters, and the patients show clinical improvement [226]. However, Valayannopoulos et al. [227] have shown that mental retardation in GA seems linked to hyperornithinemia, more than to Cre deficiency itself.

#### 6.2.3. Diagnosis

In children or adults, GA diagnosis is based mainly on ocular clinical parameters. Suspicion is confirmed with increased plasma Orn levels. Firm diagnosis remains dependent on demonstrating deficient OAT activity in fibroblasts [228].

The neonatal period is a broad time window for diagnosis. Importantly, current diagnosis is usually made at the symptomatic phase, characterized by irreversible ocular damage. Diagnosis in early infancy remains unusual, and is difficult because increased plasma ammonia and urine orotic acid, found from the first few days of life, associated with reduced levels of plasma Arg and Cit can easily lead to a diagnosis of Orn carbamoyltransferase (OTC) deficiency [228]. In order to avert or delay the appearance of symptoms, clinicians need an early diagnostic test (before clinical expression) to allow early management of the disease and a better prognosis.

Interestingly, the 3-month window just after birth is physiologically characterized by a reversed flux in the OAT reaction (P5C → Orn) (see above for details). Thus during this period, a deficit in OAT activity leads to non-toxic subnormal plasma Orn concentrations. A neonatal diagnosis, permitting prompt treatment before irreversible damage has occurred, would offer real hope for preventing ocular and neurological impairment in GA.

#### 6.2.4. Diagnosis in Newborns

**Figure 11:**
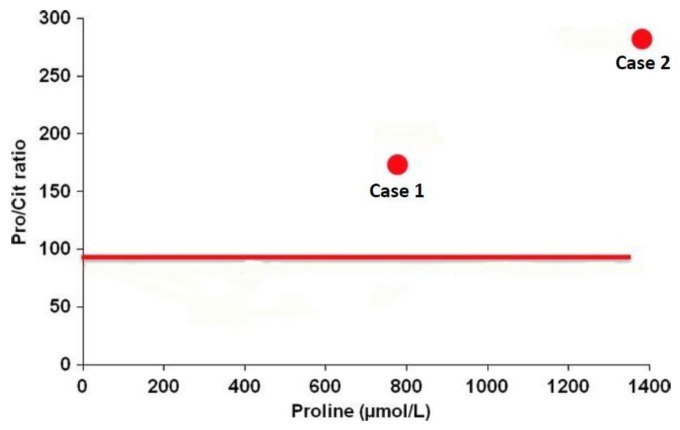
Proline/citrulline ratio and proline concentration in the neonatal dried blood spot of two affected patients with OAT deficiency (case 1 and case 2). The solid line indicates the 99 percentile (97.6) of > 450,000 blood spot samples analyzed as part of the Minnesota NBS program. Adapted from de Sain-van der Velden et al. [229].

A recent study [229] highlights the usefulness (subject to confirmation in a large-scale study) of a simple diagnostic test based on the Pro/Cit ratio in plasma and a neonatal dried blood spot (NBS). The idea of using the Pro/Cit ratio in newborns resulted from a better understanding of how OAT works at this age: in early life only, there is a reversed whole body flux of the OAT reaction in the direction of Orn synthesis [204]. OAT deficiency at this age thus theoretically causes an increase in Pro and a deficiency of Orn and its products Arg and Cit (resulting in impairment of the urea cycle and attendant hyperammonemia), and so the Pro/Cit ratio increases. In practice, concentrations of plasma Orn and Arg are often subnormal in newborns with OAT deficiency: Pro is increased, while Cit is decreased [229]. This supports the use of Pro and Cit to establish a Pro/Cit ratio with the best diagnostic sensitivity (Figure 11). OAT deficiency should be added to the differential diagnosis of hyperammonemia in newborns, and the Pro/Cit ratio, which is easy to determine, could be valuable for GA screening and diagnosis in this neonatal period.

#### 6.2.5. Therapeutic Approaches

Currently two therapeutic approaches seem to be effective to reduce plasma Orn concentrations below levels that cause toxic effects (400 µmol/L), and slow down the eye disease. These are an Arg-restricted diet [230], and vitamin B6 supplementation in cases of vitamin B6-responsive phenotype, which concerns a minority of patients [222,231]. The link between genetic mutations and response to treatment is still unclear. Data suggest that B6-responsiveness in human subjects depends on unidentified mechanisms other than specific OAT genotype [232]. This justifies trying vitamin B6 treatment in all GA patients and pursuing it if efficacious.

Concerning the treatment of mental retardation, one study [233] showed the ineffectiveness of Cre supplementation (1.5–2 g Cre monohydrate/day), consistent with involvement of plasma Orn concentration in manifesting toxic effects rather than Cre deficiency. However, the authors of this study suggest testing larger doses of Cre (as for GAMT deficiency) or initiating the treatment very early [227]. Moreover, the reduction in plasma Orn levels achieved by an Arg-restricted diet do not lead to an increase in plasma Cre levels, probably because Arg is the precursor of Cre [227].

Specifically, in children younger than 3 months of age, Arg intake should not be restricted until plasma Orn begins to increase. It is noteworthy that management of an Arg-restricted diet requires monitoring of growth, nutritional status and plasma amino acid levels.

### 6.3. OAT Inhibition As A Possible Therapeutic Strategy

#### 6.3.1. Potential Utility of OAT Inhibitors for the Treatment of Cancer

As evidenced by the live birth of OAT-null mice, OAT appears to be non-essential for cellular proliferation during embryonic and fetal development [204]. However, a study conducted by Wang et al. [123] has shown, at least for rapidly proliferating cancer cells, that OAT is needed to establish spindle formation; OAT was found to be over-expressed in some cancers, especially in HCC cells [234]. OAT inhibition could thus be beneficial in treating HCC or other cancers [34,235]. Currently, one major obstacle to the development of a drug that inhibits OAT is the fear it may give rise to toxic hyperornithinemia as observed in GA. However, GA is a slowly-progressing disease, and ocular lesions do not occur immediately. Hence acute treatments of cancer with OAT inhibitors should be possible and safe [34].

#### 6.3.2. Treatment of Hyperammonemia

Inactivation of OAT leads to an increased endogenous level of Orn with enhancement of urea formation. This effect is similar to that observed with large doses of Arg and Orn. However, OAT inhibition seems to provide a longer-lasting protective effect against various forms of hyperammonemic states. In mice with a hereditary defect of the urea cycle, OAT inhibition leads to a decrease in ammonia concentrations in plasma and tissues and a reduced excretion of urinary orotic acid [44]. No toxic effects of Orn were found in mice. Although further studies are needed to confirm this protective effect, OAT inhibition seems of interest as an approach to treating hyperammonemia.

#### 6.3.3. OAT in Sepsis

Because of its place at the crossroads of Arg and Gln synthesis, it has been suggested that OAT could play a role in the adaptive response in early endotoxemia.

Ventura et al [156] showed that the adaptive response of nitrogen metabolism in early endotoxemia is associated with a physiological inhibition of liver OAT, the latter being responsible for increased Orn in the liver. This OAT inhibition could be considered as a protective mechanism, sparing Orn for other metabolic pathways (Pro and polyamine synthesis). However, in this study, OAT seems to be non-limiting for Gln synthesis under physiological and endotoxemic conditions.

## 7. Conclusions

The importance of OAT is abundantly illustrated by the consequences of its mutation, which leads either to unviable animal newborns (unless they receive a special diet) or to gyrate atrophy, in humans. OAT is a highly conserved enzyme among eukaryotes, and is involved in amino acid metabolism. In most organisms, it is involved in the conversion of ornithine into glutamate or proline. However, in mammals, it is also involved in ornithine synthesis, depending on the organ and the development stage. This ability to switch from net ornithine degradation to net ornithine synthesis is associated with a fine regulation of its kinetic properties, and of its expression. As described above, this regulation results from the combination of several factors, ranging from the concentration of the OAT and its pH environment, to its transcription level, with a subtle balance between a background, non-specific expression, and a tissue-specific regulation by targeted transcription factors or miRNA. The huge amounts of available bioinformatics and “omics” data suggest that OAT is a player in a complex network of interacting proteins, either at the protein level or at the DNA expression level.

Although the main metabolic roles of OAT are now quite well understood, they do not explain all the reported observations—for instance, the relationship between OAT mutation and the pathophysiology of gyrate atrophy remains poorly understood. Similarly, OAT involvement in pathologies such as cancer, and its detection in the cell nucleus suggest that it could be involved in the regulation of several cell processes, probably by allowing a local control of ornithine, glutamate and related small molecules (such as polyamines, neutrotransmitters) involved in controlling the balance between the main cell function pathways.

Research is still needed to gain a better understanding of these aspects, and of how apparently unrelated observations concerning OAT can be linked.

## Figures and Tables

**Table 1:** Summary of the kinetic properties of OAT according to the tissue and the organism. Values of the Michaelis constant, *K*_m_, are given in mM.

Organ	Tissue	Species	Optimal pH	Michaelis constant (*K*_m_)		Activity		Reference
				Orn	aKG	Value	Unit	
Eye	Retina	Human	7.6–8.0	3.7	1.72	218 ± 22	nmol P5C/mg protein/h	[15]
		Bovine		5.6	1.3			[16]
		Calf				234 ± 26	nmol P5C/mg protein/h	[15]
		Rat				324 ± 43	nmol P5C/mg protein/h	[15]
		Rabbit				240 ± 24	nmol P5C/mg protein/h	[15]
	Iris (ciliary body)	Rat				308 ± 26	nmol P5C/mg protein/h	[15]
		Rabbit				227 ± 28	nmol P5C/mg protein/h	[15]
		Calf				218 ± 26	nmol P5C/mg protein/h	[15]
		Human				165 ± 17	nmol P5C/mg protein/h	[15]
Liver		Rat		2.8				[6]
						458 ± 56	nmol P5C/mg protein/h	[15]
			7.8	2.7	3.0	11	µmol/mg protein/min	[17]
		***	8.0	0.56	0.91			[18]
			8.15					[19]
				1	0.7			[14]
		Mouse	7.5	4,8	0.7	10,8	µmol/mg protein/min	[17]
		**		1.2 ± 0.11	2 ± 0.5	0,75	µmol P5C/mg proteine/h	[20]
		Rabbit				317 ± 34	nmol P5C/mg protein/h	[15]
		Trout **	7.3	7.5 ± 0.8	0.88 ± 0.2	0,033	µmol/min	[21]
	Isolated mitochondria	Rat *		4	0,15	25	nmol/min/mg protein	[22]
Kidney		Rat						
		***	8.0	0.59	0.91	920	units/mg protein	[18]
		Rabbit				674 ± 48	nmol P5C/mg protein/h	[15]
Brain		Rat				488 ± 42	nmol P5C/mg protein/h	[15]
		Rat	8	1,67	0,5			[23]
		Rabbit				112 ± 18	nmol P5C/mg protein/h	[15]
						69 ± 18	nmol P5C/mg protein/h	[15]
		Mouse **		1.1 ± 0.1	2.6	1.1	µmol P5C/mg protein/h	[20]
	Cortical interneurons	Mouse *		0.8 ± 0.3				[24]
	Cerebellar granule cells	Mouse *		4.7 ± 0.9				[24]
	Astrocytes	Mouse *		4.3 ± 2.2				[24]
Small intestine		Rat ***	8.0	0.60	0.95			[18]
								
		*Modiolus demissus*						[25]
		*Plasmodium falciparum*		3.95 ± 1.04	0.65 ± 0.21			[26]
		Pea (*Pisum sativorum*)	8.8	15	2	60.4	nmol/mg/s	[27]
		Mothbean (*Vignia aconitifolia*)	8	2	0.75			[28]

* unpurified enzyme, ** partial purification, *** crystallized enzyme.

**Table 2:** OAT inhibitors described for mammalian enzyme.

Inhibitor	Structure	Nature	Experimental Conditions	*K*_i_ or % Inhibition	OAT Origin	Reference
***α-Amino acids***						
l-Canaline	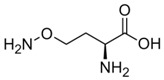	Irreversible	8 U OAT, 37 °C, pH 8, 0.05 mM PO_4_^3−^ buffer 0.1 mM PLP, 175 mM Orn, 35 mM aKG	*K*_i_: 2 µM	Rat (liver)	[39]
		Irreversible, noncompetitive	pH 8, 0.05 mM PO_4_^3−^ buffer 0.01 mM PLP, 20 mM Orn, 10 mM aKG		Rat (liver)	[40]
l-Canavanine	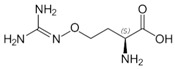		37 °C, pH 7.1, 50 mM PO_4_^3−^ buffer 35 mM Orn, 3.75 mM aKG,K	100% at 25 mM	Rat (liver)	[6]
			0.5 U OAT; 50 mM Orn	23% at 20 mM	Rat (kidney)	[41]
l-Norvaline	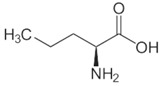		37 °C, pH 7.1, 50 mM PO_4_^3−^ buffer 35 mM Orn, 3.75 mM aKG,K	59% at 50 mM	Rat (liver)	[6]
			pH 8, 50 mM PO_4_^3−^ buffer 0.1 mM PLP, 10 mM Orn, 5 mM aKG	50% at 10 mM	Rat (liver)	[14]
			0.5 U OAT; 50 mM Orn	44% at 100 mM	Rat (kidney)	[41]
l-Valine	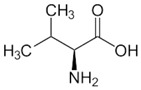		37 °C, pH 7.1, 50 mM PO_4_^3−^ buffer 35 mM Orn, 3.75 mM aKG,K	47% at 25 mM	Rat (liver)	[6]
			0.5 U OAT; 50 mM Orn	33% at 25 mM	Rat (kidney)	[41]
		Competitive for Orn; uncompetitive for aKG	pH 8, 50 mM PO_4_^3−^ buffer 0.1 mM PLP, 10 mM Orn, 5 mM aKG	2.0 mM (*forward reaction*)	Rat (liver)	[14]
		Noncompetitive for Glu and P5C	37 °C, pH 8, 50 mM Tris-HCl buffer 0.1 mM PLP, 5 mM P5C, 40 mM l-Glu	20 mM (*reverse reaction*)	Rat (liver)	[14]
		Competitive	pH 8, 12.5 mM Tris-HCl buffer 0.025 mM PLP, 5 mM Orn, 2.5 aKG	28% at 2.5 mM 41% at 5 mM	Rat (brain)	[23]
l-Isoleucine	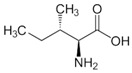		37 °C, pH 7.1, 50 mM PO_4_^3−^ buffer 35 mM Orn, 3.75 mM aKG,K	27% at 25 mM	Rat (liver)	[6]
			pH 8, 50 mM PO_4_^3−^ buffer 0.1 mM PLP, 10 mM Orn, 5 mM aKG	30% at 10 mM	Rat (liver)	[14]
			0.5 U OAT; 50 mM Orn	19% at 25 mM	Rat (kidney)	[41]
			pH 8, 12.5 mM Tris-HCl buffer 0.025 mM PLP, 5 mM Orn, 2.5 aKG	29% at 2.5 mM 29% at 5 mM	Rat (brain)	[23]
l-Leucine	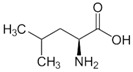		37 °C, pH 7.1, 50 mM PO_4_^3−^ buffer 35 mM Orn, 3.75 mM aKG,K	21% at 25 mM	Rat (liver)	[6]
			pH 8, 50 mM PO_4_^3−^ buffer 0.1 mM PLP, 10 mM Orn, 5 mM aKG	20% at 10 mM	Rat (liver)	[14]
			0.5 U OAT; 50 mM Orn	17% at 25 mM	Rat (kidney)	[41]
			pH 8, 12.5 mM Tris-HCl buffer 0.025 mM PLP, 5 mM Orn, 2.5 aKG	8% at 2.5 mM 22% at 5 mM	Rat (brain)	[23]
Cysteine			pH 8, 12.5 mM Tris-HCl buffer 0.025 mM PLP, 5 mM Orn, 2.5 aKG	48% at 2.5 mM 66% at 5 mM	Rat (brain)	[23]
2,4-Diaminobutyrate	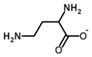	Competitive	37 °C, pH 8, 50 mM PO_4_^3−^ buffer 175 mM Orn, 35 mM aKG	25% at 10 mM 40% at 25 mM	Rat (liver)	[42]
***γ-Amino acids***						
γ-Aminobutyrate	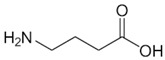	Competitive	37 °C, pH 8, 50 mM PO_4_^3−^ buffer 175 mM Orn, 35 mM aKG	3.4 mM 70% at 10 mM 90% at 25 mM	Rat (liver)	[42]
			37 °C, pH 7.1, 50 mM PO_4_^3−^ buffer 35 mM Orn, 3.75 mM aKG,K	47% at 25 mM	Rat (liver)	[6]
			pH 8, 50 mM PO_4_^3−^ buffer 0.1 mM PLP, 10 mM Orn, 5 mM aKG	26% at 10 mM	Rat (liver)	[14]
(GABA)			0.5 U OAT; 50 mM Orn	26% at 25 mM	Rat (kidney)	[41]
5-Aminovalerate	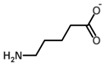		37 °C, pH 7.1, 50 mM PO_4_^3−^ buffer 35 mM Orn, 3.75 mM aKG,K	100% at 25 mM	Rat (liver)	[6]
(δ-Aminovalerate)			0.5 U OAT; 50 mM Orn	15% at 25 mM	Rat (kidney)	[41]
		Competitive for Orn	pH 8, 0.05 mM PO_4_^3−^ buffer 0.01 mM PLP, 20 mM Orn, 10 mM aKG	17.7 mM	Rat (liver)	[40]
ε-Aminocaproate	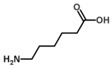		0.5 U OAT; 50 mM Orn	6% at 25 mM	Rat (kidney)	[41]
***α-Ketoacids***						
α-Ketovalerate	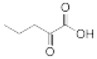		37 °C, pH 7.1, 50 mM PO_4_^3−^ buffer 35 mM Orn, 3.75 mM aKG,K	66% at 21 mM	Rat (liver)	[6]
			0.5 U OAT; 50 mM Orn	38% at 25 mM	Rat (kidney)	[41]
α-Ketoisocaproate	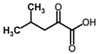		37 °C, pH 7.1, 50 mM PO_4_^3−^ buffer 35 mM Orn, 3.75 mM aKG,K	60% at 19 mM	Rat (liver)	[6]
(Ketoleucine)			0.5 U OAT; 50 mM Orn	41% at 25 mM	Rat (kidney)	[41]
α-Ketoisovalerate	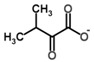		37 °C, pH 7.1, 50 mM PO_4_^3−^ buffer 35 mM Orn, 3.75 mM aKG,K	45% at 22 mM	Rat (liver)	[6]
			0.5 U OAT; 50 mM Orn	34% at 25 mM	Rat (kidney)	[41]
α-Ketocaproate	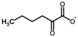		37 °C, pH 7.1, 50 mM PO_4_^3−^ buffer 35 mM Orn, 3.75 mM aKG,K	18% at 15 mM	Rat (liver)	[6]
			0.5 U OAT; 50 mM Orn	34% at 25 mM	Rat (kidney)	[41]
α-Ketobutyrate	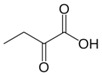		37 °C, pH 7.1, 50 mM PO_4_^3−^ buffer 35 mM Orn, 3.75 mM aKG,K	13% at 10 mM	Rat (liver)	[6]
			0.5 U OAT; 50 mM Orn	27% at 25 mM	Rat (kidney)	[41]
***Polyamines***						
Cadaverine	H_2_N-(CH_2_)_5_-NH_2_		37 °C, pH 7.1, 50 mM PO_4_^3−^ buffer 35 mM Orn, 3.75 mM aKG,K	14% at 25 mM	Rat (liver)	[6]
			0.5 U OAT; 50 mM Orn	5.7% at 25 mM	Rat (kidney)	[41]
Putrescine	H_2_N-CH_2_-CH_2_-CH_2_-CH_2_-NH_2_		37 °C, pH 7.1, 50 mM PO_4_^3−^ buffer 35 mM Orn, 3.75 mM aKG,K	14% at 25 mM	Rat (liver)	[6]
			pH 8, 50 mM PO_4_^3−^ buffer 0.1 mM PLP, 10 mM Orn, 5 mM aKG	*No inhibition* at 10 mM	Rat (liver)	[14]
			0.5 U OAT; 50 mM Orn	3% at 25 mM	Rat (kidney)	[41]
			pH 8, 12.5 mM Tris-HCl buffer 0.025 mM PLP, 5 mM Orn, 2.5 aKG	*No inhibition* at 5 mM 8% at 10 mM	Rat (brain)	[23]
Spermine	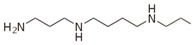		pH 8, 12.5 mM Tris-HCl buffer 0.025 mM PLP, 5 mM Orn, 2.5 aKG	33% at 5 mM 56% at 10 mM	Rat (brain)	[23]
			pH 8, 50 mM PO_4_^3−^ buffer 0.1 mM PLP, 10 mM Orn, 5 mM aKG	*No inhibition* at 10 mM	Rat (liver)	[14]
Spermidine	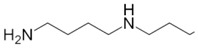		pH 8, 12.5 mM Tris-HCl buffer 0.025 mM PLP, 5 mM Orn, 2.5 aKG	30% at 5 mM 48% at 10 mM	Rat (brain)	[23]
Histamine	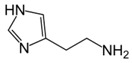		pH 8, 12.5 mM Tris-HCl buffer 0.025 mM PLP, 5 mM Orn, 2.5 aKG	42% at 5 mM 50% at 10 mM	Rat (brain)	[23]
***Other inhibitors***						
5-Fluoromethylornithine	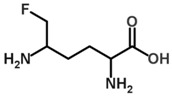	Irreversible	37 °C, pH 8, 50 mM PO_4_^3−^ buffer 175 mM Orn, 35 mM aKG	70 µM	Rat (liver; partially purified)	[37]
			8 U OAT, 37°C, pH 8, 0.05 mM PO_4_^3−^ buffer 0.1 mM PLP, 175 mM Orn, 35 mM aKG	30 µM	Rat (liver)	[39]
			*Not given*	11 ± 2 µM		[36]
*S*,*S*-5-Fluoromethylornithine			*Not given*	2.4 ± 0.3 µM		[36]
*N*-Acetylornithine	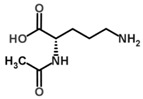	Competitive with Orn	pH 8, 0.05 mM PO_4_^3−^ buffer 0.01 mM PLP, 20 mM Orn, 10 mM aKG	8.8 mM	Rat (liver)	[40]
			pH 8, 12.5 mM Tris-HCl buffer 0.025 mM PLP, 5 mM Orn, 2.5 aKG	24% at 2.5 mM 27% at 5 mM	Rat (brain)	[23]
4-Aminohexynoate	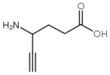	Irreversible	0.5 U OAT, 37 °C, 40 mM KPP0,020 mM PLP, 60 mM Orn, 21 mM aKG		Rat (liver)	[43]
Gabaculine	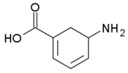	Irreversible	0.5 U OAT, 37 °C, 40 mM KPP 0,020 mM PLP, 60 mM Orn, 21 mM aKG		Rat (liver)	[43]
2-aminooxyacetate	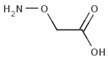			“almost complete” at 0.2 mM	Rat	[44]

**Table 3:** OAT activity measured in various tissues, with no enzyme isolation.

Organ	Tissue	Species	Activity	Unit	Reference
Eye	Neuroretina	Cat	88.2	mmol/kg dry wt/h	[70]
		Ox	71 ± 17	nmol/mg protein/30 min	[132]
	Optic nerve	Cat	6.7	mmol/kg dry wt/h	[70]
	Tapetum	Cat	26.7	mmol/kg dry wt/h	[70]
	Sclera	Cat	5.36	mmol/kg dry wt/h	[70]
	Choroid	Cat	17.4	mmol/kg dry wt/h	[70]
		Ox	14 ± 5	nmol/mg protein/30 min	[132]
	Retina	Rat	174 ± 25	nmol/mg protein/h	[133]
			97 ± 9	nmol/mg protein/30 min	[132]
		Ox	119 ± 34	nmol/mg protein/h	[133]
		Pig	409 ± 50	nmol/mg protein/h	[133]
		Dog	159	nmol/mg protein/h	[133]
		Chicken	124 ± 16	nmol/mg protein/h	[133]
			120 ± 20	nmol/mg/h	[134]
		Turkey	85	nmol/mg protein/h	[133]
		*Rana pipiens*	302	nmol/mg protein/h	[133]
		*Rana clamitans*	238	nmol/mg protein/h	[133]
		*Xenopus laevis*	392 ± 93	nmol/mg protein/h	[133]
		*Cyprinus carpio*	766 ± 57	nmol/mg protein/h	[133]
	Pigment epithelium (iris epithelium)	Cat	138	mmol/kg dry wt/h	[70]
		Rat	988 ± 130	nmol/mg protein/h	[133]
		Ox	497 ± 89	nmol/mg protein/h	[133]
			113 ± 24	nmol/mg protein/30 min	[132]
		Pig	2260 ± 630	nmol/mg protein/h	[133]
		Dog	602 ± 159	nmol/mg protein/h	[133]
		Chicken	1431 ± 535	nmol/mg protein/h	[133]
			1670 ± 55	nmol/mg/h	[134]
		Turkey	1150	nmol/mg protein/h	[133]
		*Rana pipiens*	795	nmol/mg protein/h	[133]
		*Rana clamitans*	217	nmol/mg protein/h	[133]
		*Xenopus laevis*	171 ± 87	nmol/mg protein/h	[133]
		*Cyprinus carpio*	203 ± 51	nmol/mg protein/h	[133]
	Vascular layer of the ciliary body	Cat	63.0	mmol/kg dry wt/h	[70]
	Cornea	Cat	8.11	mmol/kg dry wt/h	[70]
		Ox	14 ± 9	nmol/mg protein/30 min	[132]
	Lens	Cat	No		[70]
		Ox	No		[132]
Brain	Frontal cortex	Rat	1.45 ± 0.29	mU/mg protein	[135]
	Occipital cortex	Rat	1.38 ± 0.08	mU/mg protein	[135]
	Olfactory bulb	Rat	1.23 ± 0.07	mU/mg protein	[135]
	Hippocampus	Rat	1.33 ± 0.16	mU/mg protein	[135]
	Striatum	Rat	0.95 ± 0.06	mU/mg protein	[135]
	Midbrain, thalamus	Rat	1.17 ± 0.12	mU/mg protein	[135]
	Hypothalamus	Rat	1.34 ± 0.10	mU/mg protein	[135]
	Pons, medulla oblongata	Rat	0.85 ± 0.04	mU/mg protein	[135]
	Cerebellum	Rat	0.90 ± 0.19	mU/mg protein	[135]
	Tractus opticus	Rat	0.75 ± 0.10	mU/mg protein	[135]
	Spinal cord	Rat	0.60 ± 0.07	mU/mg protein	[135]
Liver		Rat	179	nmol/mg protein/h	[133]
			242 ± 41	nmol/mg protein/30 min	[132]
		Pig	164	nmol/mg protein/h	[133]
		Ox	12	nmol/mg protein/30 min	[132]
		Chicken	174 ± 22	nmol/mg protein/h	[133]
			150 ± 40	nmol/mg/h	[134]
		*Rana clamitans*	137	nmol/mg protein/h	[133]
		*Xenopus laevis*	161 ± 101	nmol/mg protein/h	[133]
		*Cyprinus carpio*	590	nmol/mg protein/h	[133]
